# Regulation of Neutrophilic Inflammation by Proteinase-Activated Receptor 1 during Bacterial Pulmonary Infection

**DOI:** 10.4049/jimmunol.1500124

**Published:** 2015-05-06

**Authors:** Ricardo J. José, Andrew E. Williams, Paul F. Mercer, Michal G. Sulikowski, Jeremy S. Brown, Rachel C. Chambers

**Affiliations:** Centre for Inflammation and Tissue Repair, University College London, London WC1E 6JF, United Kingdom

## Abstract

Neutrophils are key effector cells of the innate immune response to pathogenic bacteria, but excessive neutrophilic inflammation can be associated with bystander tissue damage. The mechanisms responsible for neutrophil recruitment to the lungs during bacterial pneumonia are poorly defined. In this study, we focus on the potential role of the major high-affinity thrombin receptor, proteinase-activated receptor 1 (PAR-1), during the development of pneumonia to the common lung pathogen *Streptococcus pneumoniae.* Our studies demonstrate that neutrophils were indispensable for controlling *S. pneumoniae* outgrowth but contributed to alveolar barrier disruption. We further report that intra-alveolar coagulation (bronchoalveolar lavage fluid thrombin–antithrombin complex levels) and PAR-1 immunostaining were increased in this model of bacterial lung infection. Functional studies using the most clinically advanced PAR-1 antagonist, SCH530348, revealed a key contribution for PAR-1 signaling in influencing neutrophil recruitment to lung airspaces in response to both an invasive and noninvasive strain of *S. pneumoniae* (D39 and EF3030) but that PAR-1 antagonism did not impair the ability of the host to control bacterial outgrowth. PAR-1 antagonist treatment significantly decreased pulmonary levels of IL-1β, CXCL1, CCL2, and CCL7 and attenuated alveolar leak. Ab neutralization studies further demonstrated a nonredundant role for IL-1β, CXCL1, and CCL7 in mediating neutrophil recruitment in response to *S. pneumoniae* infection. Taken together, these data demonstrate a key role for PAR-1 during *S. pneumoniae* lung infection that is mediated, at least in part, by influencing multiple downstream inflammatory mediators.

## Introduction

Lower respiratory tract infections are a leading cause of morbidity and mortality and represent an enormous global healthcare burden (1). Infection with *Streptococcus pneumoniae* is the predominant cause of pneumonia and consequently one of the most common causes of death associated with infectious disease worldwide (2, 3). Bacterial pneumonia in humans is often accompanied by a pronounced inflammatory response, characterized by an acute phase reaction, the release of proinflammatory mediators, a heightened procoagulant state, and the recruitment of large numbers of neutrophils to the lung (4–6). This response is important for the control of bacterial infection (7–10), but excessive neutrophilic inflammation can also lead to bystander tissue damage, characterized by disruption of the alveolar barrier, pulmonary edema, and severely compromised lung function (11, 12).

The local activation of coagulation within the intra-alveolar compartment is a key feature of the pulmonary response to injury and infection (13). The major high-affinity thrombin receptor, proteinase-activated receptor 1 (PAR-1), has been shown to play a central role in mediating the interplay between coagulation and inflammation. PAR-1 activation is initiated via the proteolytic unmasking of an N-terminal tethered ligand, which in turn binds to the second extracellular loop to initiate cell signaling via the recruitment of heterotrimeric G proteins (13, 14). PAR-1 signaling has been shown to contribute to the pathogenesis of pulmonary fibrosis and acute lung injury in experimental models (15, 16). However, the contribution of PAR-1 signaling during pulmonary bacterial infection remains poorly defined, particularly during the acute phase of the neutrophilic inflammatory response to *S. pneumoniae*.

To investigate the extent that neutrophils contribute to alveolar barrier disruption during pulmonary bacterial infection, we used a mouse model of *S. pneumoniae* challenge. The interplay between PAR-1, neutrophilic inflammation, and alveolar leak was investigated using a potent, highly selective and clinically advanced PAR-1 antagonist, SCH530348 (17–19), in mice challenged with both an invasive and noninvasive strain of *S. pneumoniae*. To characterize the inflammatory mediators that may be regulating neutrophil recruitment and pulmonary inflammation downstream of PAR-1 signaling, we measured several proinflammatory cytokines and chemokines following PAR-1 antagonist treatment. Ab neutralization studies confirmed that several cytokines and chemokines were involved in regulating neutrophil recruitment during *S. pneumoniae* infection. Finally, the effect of PAR-1 antagonism on the control of bacterial outgrowth was also investigated. Taken together, our findings reveal a key role for PAR-1 in influencing neutrophil recruitment and alveolar barrier disruption during pneumococcal pneumonia, with potential important implications for the treatment of excessive inflammation in this disease setting.

## Materials and Methods

### Ethics statement

All animal studies were performed according to the UK Home Office Animals Scientific Procedures Act. Mice were kept in a specified pathogen-free facility at University College London and housed in individually ventilated cages, with free access to food and water.

### Bacterial growth conditions and culture

*S. pneumoniae* (serotype 2 [D39] or 19F [EF3030]) was cultured on plates containing Columbia agar (Oxoid, Basingstoke, U.K.) and 5% defribrinated horse blood (TCS Biosciences, Buckingham, U.K.) or in Todd–Hewitt medium (THY, Oxoid) containing 5% yeast extract at 37°C in the presence of 5% CO_2_. Growth in medium was assessed by measuring the OD at 580 nm with a spectrophotometer (Amersham Pharmacia Biotech, Little Chalfont, U.K.). Bacterial stocks were grown to midlog phase (OD at 580 nm, 0.4–0.5) before being mixed in 10% glycerol and frozen at −80°C in single-use aliquots. Bacterial counts (CFU) were calculated by plating serial dilutions of the bronchoalveolar lavage (BAL) fluid, whole-lung homogenate suspensions, or blood onto blood agar plates and incubated for 18 h at 37°C with 5% CO_2_.

### Bacterial strain, infection model, cell isolation, and analysis

Female BALB/c mice (Harlan Laboratories, Bicester, U.K.), 7–8 wk of age, were anesthetized with 5% isoflurane and each mouse was inoculated intranasally with 50 μl containing 5 × 10^6^ CFU *S. pneumoniae* serotype 2 (D39 strain), unless otherwise stated. In confirmatory experiments of the effect of SCH530348 on host defense, mice were challenged with *S. pneumoniae* 19F (EF3030). Mice were culled at 4, 24, or 48 h and BAL fluid, whole lungs, and blood were removed postmortem. BAL fluid, whole-lung homogenates, and blood samples were collected and prepared for analysis as previously described (16, 20).

### PAR-1 antagonists and in vivo infection model

The potent and highly selective PAR-1 antagonist, SCH530348, a synthetic tricyclic 3-phenylpyridine analog of himbacine with the chemical name of ethyl[(*R*1*R*,3a*R*,4a*R*,6*R*,8a*R*,9*S*,9a*S*)-9-[(*E*)-2-[5-(3-fluorophenyl)-2-pyridinyl]ethenyl]-dodecahydro-1-methyl-3-oxonaphtho[2,3-c]furan-6-yl]carbamate was originally developed by Shering-Plough (17). Compound for all studies was sourced from Axon Medchem (Groningen, The Netherlands) as product number 1755. The compound was dissolved in vehicle (30% Solutol; macrogol 15 hydroxystearate, polyoxyl 15 hydroxystearate [Kolliphor HS 15, Sigma-Aldrich, Dorset, U.K.] in sterile double distilled H_2_O) and the dose of SCH530348 was determined according to published pharmacokinetic data in rodent models (17).

RWJ58259 is a specific PAR-1 antagonist (α*S*)-*N*-[(1*S*)-3-amino-1-[[(phenylmethyl)amino]carbonyl]propyl]-α-[[[[1-(2,6-dichlorophenyl)methyl]-3-(1-pyrrolidinylmethyl)-1*H*-indazol-6-yl]amino]carbonyl]amino]-3,4-difluorobenzenepropanamide and the dose was used as previously described (16).

Mice were challenged with an intranasal inoculation of *S. pneumoniae* (D39 or EF3030; ∼5 × 10^6^ CFU) or sterile PBS and immediately treated with vehicle (30% Solutol; macrogol 15 hydroxystearate, polyoxyl 15 hydroxystearate [Kolliphor HS 15, Sigma-Aldrich] in sterile double distilled H_2_O) or the PAR-1 antagonist SCH530348 (10 mg/kg) in vehicle by i.p. injection. The number of bacteria administered was determined by plating the inoculum used for each experiment. In separate experiments, mice were treated with the vehicle or the PAR-1 antagonist every 12 h and culled at either 4, 24, or 48 h, and BAL fluid, whole-lung tissue, and blood were removed postmortem.

### Immunohistochemistry and image analysis

The immunohistochemistry method was followed as described previously (16). Briefly, mouse lungs were inflated with 10% neutral buffered formalin (Sigma-Aldrich) and fixed for 24 h at room temperature in 10% neutral buffered formalin. Serial paraffin-embedded sections (4 μm) were de-waxed and Ags were unmasked by microwaving sections with 10 mM sodium citrate buffer (pH 6.0) for PAR-1 and *S. pneumoniae* staining. Ag retrieval was omitted for the Ly6G neutrophil-specific marker. Endogenous peroxide activity was blocked with 3% hydrogen peroxide (Sigma-Aldrich) and sections were also blocked with 3% goat serum with 1% BSA (Merck, Darmstadt, Germany) in PBS prior to incubation with primary Abs. Primary Abs to PAR-1 (0.8 μg/ml, Santa Cruz Biotechnology, sc-5605), Ly6G (2 μg/ml, BD Biosciences, 551459), and pneumococcal anti-serotype 2 D39 (dilution 1:20,000, Staten Institute, 16745) were incubated for 16 h at 4°C. IgG controls (Vector Laboratories, Orton Southgate, U.K.) were included as a negative control. The following day, sections were incubated for 30 min with biotin-labeled secondary Abs (Vector laboratories) followed by 30 min incubation with ABC complex (Vector Laboratories). Color was developed with diaminobenzidine chromogen (BioGenex) for 5 min. Sections were counterstained with Gill II hematoxylin, dehydrated, and permanently coverslipped. Slides were digitally scanned using a NanoZoomer (Hamamatsu) at the ×20 objective size. Automatic quantitation of immunostained tissue sections was performed using Definiens Tissue Studio (version 3.6). Three algorithms were set up for each of the Ab markers used based on the chromogenic diaminobenzidine staining pattern, including 1) positive index for anti-Ly6G Ab staining (neutrophils) defined as the ratio of all positively stained neutrophils detected by immunohistochemistry to all nuclei stained with blue hematoxylin for each individual scan; 2) percentage of anti–*S. pneumoniae* capsular serotype 2 Ab staining; and 3) percentage and intensity of anti–PAR-1 immunostaining (classified as weak, medium, and high). The magnification and resolution of digitally scanned sections with the Hamamatsu NanoZoomer (.ndp file) was automatically read by the Definiens software. The analysis was blinded to treatment groups throughout this analysis. Image analysis was carried out using the Definiens algorithm corresponding to a ×10 objective, which created the grid on the whole scans and defined each tile of the heat maps. A ×10 objective was employed to provide an ideal ratio between image resolution and lung area sampled. The tiles that did not contain tissue (outside of region of interest) were excluded from the analysis.

### Neutrophil depletion

Ly6G^+^ neutrophils were depleted as previously described (21). Briefly, 600 μg anti-Ly6G mAb (clone 1A8, Bio X Cell) was administered by i.p. injection in a volume of 200 μl 24 h prior to *S. pneumoniae* challenge. Control mice were given 600 μg isotype control IgG2 Ab (clone 2A3, Bio X Cell). Neutrophil depletion was confirmed by counting cells in the BAL fluid and by demonstrating with flow cytometry a marked reduction in anti-GR1^+^ cells in whole-lung homogenates (data not shown) of mice challenged with *S. pneumoniae*.

### Protein, cytokine, and chemokine analysis

Analysis of endothelial–epithelial barrier permeability was performed by measuring mouse serum albumin in recovered BAL fluid by ELISA according to the manufacturer’s instructions (Bethyl Laboratories). Coagulation activation was determined by measuring thrombin–antithrombin complexes (TAT) in BAL fluid by ELISA (EIAab). Cytokines (IFN-γ, IL-1β, IL-6, IL-10, and TNF) and chemokine (KC/Gro) were measured with the mouse proinflammatory 7-plex ultra-sensitive kit (Meso Scale Discovery, Rockville, MD) according to the manufacturer’s protocol. The chemokine CCL2 (MCP-1) was measured by ELISA (R&D Systems Europe) or the mouse CCL2 ultra-sensitive kit (Meso Scale Discovery) according to the manufacturer’s protocol, and CCL7 (MCP-3) was measured by ELISA (PromoKine) according to the manufacturer’s protocol.

### In vivo neutralization of bronchoalveolar cytokines and chemokines

Mice were intranasally challenged with sterile PBS or *S*. *pneumoniae* D39 (5 × 10^6^ CFU, 50 μl). Additionally, the inoculum contained 10 μg neutralizing Ab per mouse (anti-CXCL1, PeproTech, London, U.K.; anti-CCL2, R&D Systems Europe; anti-CCL7, PeproTech; or anti–IL-1β,Thermo Fisher Scientific, Leicestershire, U.K.) or poly-IgG control (PeproTech) (16). Additionally, in a separate experiment anti-CXCL1 (PeproTech) was administered i.p. 2 h before *S. pneumoniae* challenge as previously described (22). Animals were culled at 4 h postchallenge and BAL fluid, whole-lung tissue, and blood were removed postmortem.

### Statistical analysis

Data were analyzed using Graphpad Prism version 6. Data were statistically compared by a Student *t* test for the comparison of two groups. The comparison of more than two groups was made by one-way or two-way ANOVA with a Holm–Sidak post hoc test as appropriate. Correlations were analyzed with Pearson correlation coefficients. All *p* values are two-tailed and were considered significant when *p* < 0.05.

## Results

### Neutrophil recruitment is required for host defense to *S. pneumoniae* but disrupts alveolar barrier integrity

**FIGURE 1.**
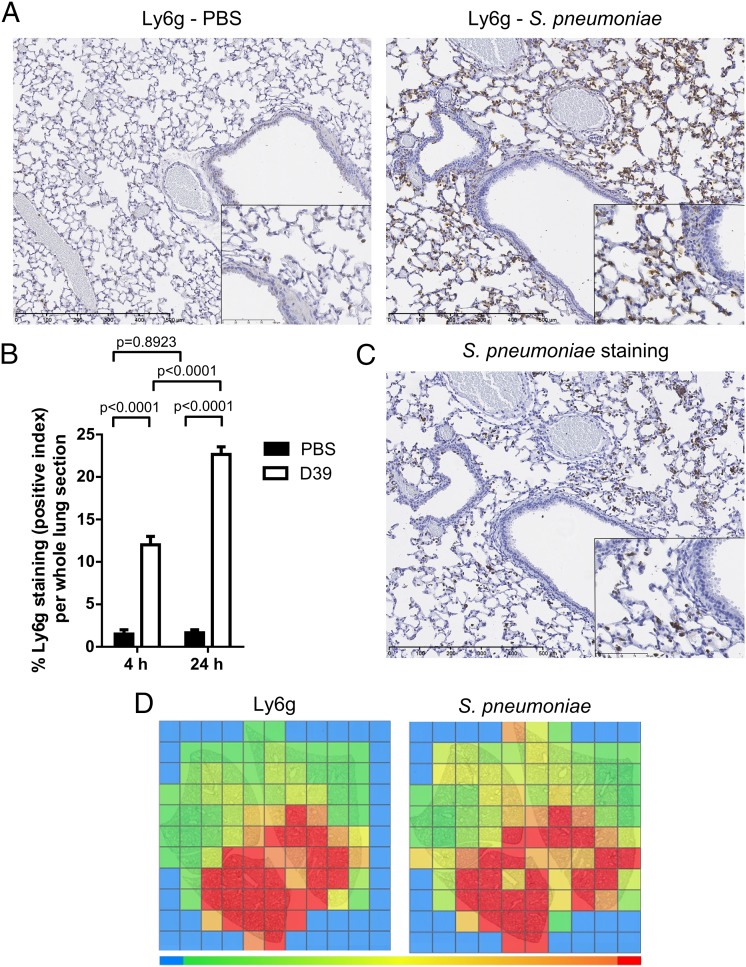
Characterization of neutrophil recruitment and *S. pneumoniae* infection in BALB/c mice. BALB/c mice (7–8 wk old) were challenged intranasally with *S. pneumoniae* D39 (5 × 10^6^ CFU) or PBS and culled at 4 h and 24 h for (**A**) immunohistochemistry of lung sections at 24 h stained with a specific Ly6G Ab and counterstained with hematoxylin. Original magnification ×10; *inset,* ×40. (**B**) Quantification of Ly6G staining at 4 and 24 h on lung sections from PBS-challenged and *S. pneumoniae*–infected mice. (**C**) Immunohistochemistry for *S. pneumoniae* serotype 2 on lung sections at 24 h from mice challenged with *S. pneumoniae* (original magnification ×10; *inset,* ×40, counterstained with hematoxylin) and (**D**) heat maps representative of Ly6G and *S. pneumoniae* staining intensity at 24 h. Data are expressed as bar graphs with means ± SEM (*n* = 6/group) and analyzed with the unpaired Student *t* test and two-way ANOVA with a Holm–Sidak post hoc test.

We first characterized the neutrophilic response during bacterial infection in our model of *S. pneumoniae* pneumonia (serotype 2; D39, 5 × 10^6^ CFU/ml intranasally, in BALB/c female mice 7–8 wk old). Immunohistochemical analysis of Ly6G^+^ neutrophils revealed that neutrophil accumulation was significantly increased at the earliest time point examined (4 h) and was further increased 24 h postinfection (Fig. 1A, 1B). Furthermore, we performed immunostaining for *S. pneumoniae* (pneumococcal anti-serotype 2, D39) to determine the colocalization of bacteria with neutrophils (Fig. 1C). Heat maps of immunostaining intensity generated by quantitative image analysis (based on algorithms generated using Definiens software) of multiple serial sections confirmed that neutrophils were recruited predominantly to sites of *S. pneumoniae* localization (Fig. 1D).

**FIGURE 2.**
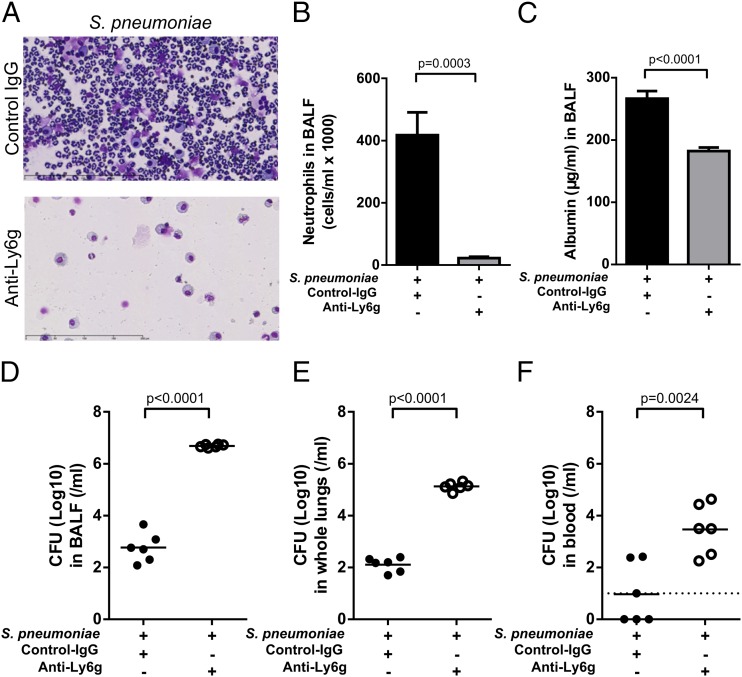
Neutrophil depletion protects mice from alveolar leak but compromises host defense. BALB/c mice (7–8 wk old) were treated with anti-Ly6G or anti-IgG (control Ab) 24 h prior to being challenged intranasally with *S. pneumoniae* D39 (5 × 10^6^ CFU). Mice were culled at 24 h postinfection. (**A**) Representative cytospins of cells recovered from BAL fluid (Romanowski stain; original magnification ×40) and (**B**) neutrophil counts. (**C**) Albumin levels recovered from BAL fluid 24 h following infection were measured as a marker of alveolar leak. Bacterial CFU recovered from (**D**) BAL fluid, (**E**) lung homogenates, and (**F**) blood were measured 24 h following infection.

We next determined the contribution of neutrophil recruitment to alveolar barrier disruption following *S. pneumoniae* challenge. Mice were injected with an anti-Ly6G Ab to deplete neutrophils prior to lung infection. The number of neutrophils recovered from BAL fluid at 24 h was reduced by 95% compared with *S. pneumoniae*–infected mice treated with a control IgG Ab (Fig. 2A, 2B). Neutrophil depletion was accompanied by a significant reduction in BAL fluid albumin levels compared with IgG control Ab–treated mice (Fig. 1C). These albumin levels were similar to the levels measured in PBS only–challenged mice (mean ± SEM, 190 ± 18.87 μg), indicating that neutrophils contribute to the disruption of the alveolar barrier during *S. pneumoniae* infection. However, neutrophil depletion also resulted in increased *S. pneumoniae* CFU recovered from the BAL fluid, whole-lung homogenates, and blood (Fig. 2D–F), indicating that neutrophils play an indispensable role in controlling bacterial outgrowth in this model.

### PAR-1 expression and coagulation activation are increased following *S. pneumoniae* infection

**FIGURE 3.**
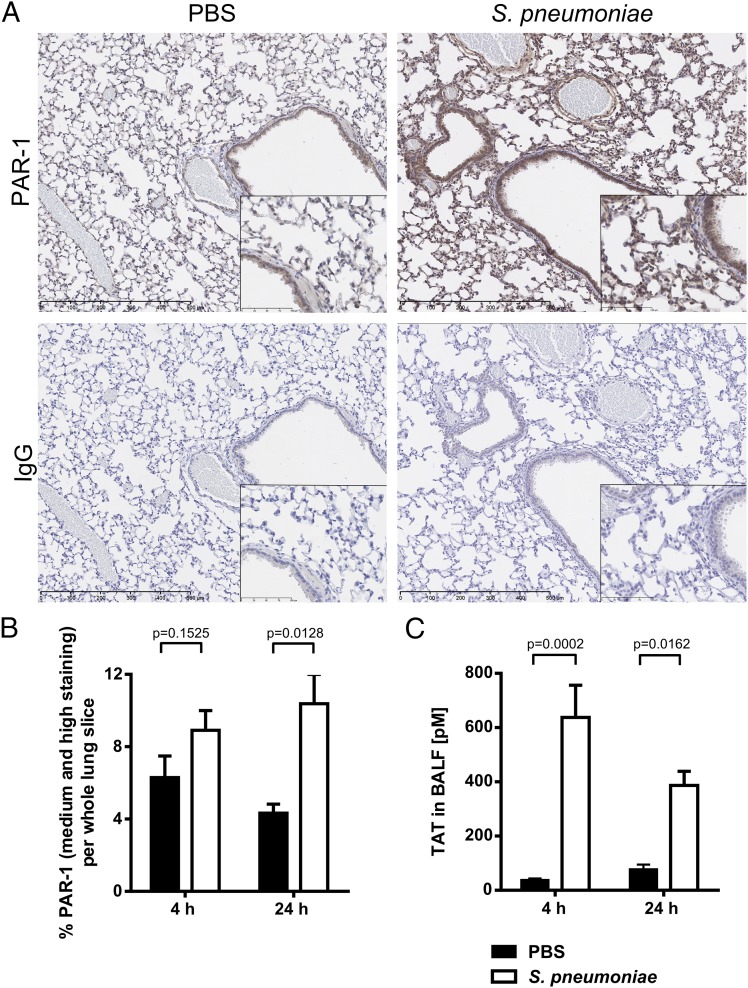
PAR-1 expression and coagulation activation is increased following *S. pneumoniae* infection. BALB/c mice (7–8 wk old) were challenged intranasally with *S. pneumoniae* D39 (5 × 10^6^ CFU) or PBS and culled at 4 h or 24 h. (**A**) Immunohistochemistry staining for PAR-1 on lung sections from mice challenged with PBS and *S. pneumoniae* 24 h postinfection (original magnification ×10; *inset,* ×40; IgG, negative control; counterstained with hematoxylin). (**B**) Quantification of PAR-1 staining intensity in lung sections at 4 and 24 h for mice challenged with PBS or infected with *S. pneumoniae*. (**C**) TAT complexes were measured by ELISA. Data are expressed as bar graphs with means ± SEM (*n* = 3–6/group) and analyzed with two-way ANOVA with a Holm–Sidak post hoc test.

We next investigated whether PAR-1 is involved in the host response to *S. pneumoniae* infection. We first examined PAR-1 expression by immunohistochemistry in serial lung sections. PAR-1 immunostaining in the lung at baseline was prominent for multiple cell types, including bronchiolar and alveolar epithelium, endothelium, and macrophages (Fig. 3A). PAR-1 staining intensity was increased in *S. pneumoniae*–infected mice at 24 h. Increased PAR-1 expression in *S. pneumoniae*–infected mice at 24 h was confirmed by quantitative image analysis (based on algorithms generated using Definiens software) (Fig. 3B).

Because PAR-1 is the major high-affinity receptor for thrombin and other key enzymes of the extrinsic coagulation cascade, we next assessed coagulation activation during *S. pneumoniae* infection by measuring TAT levels in BAL fluid. *S. pneumoniae* infection significantly increased TAT levels at 4 and 24 h (Fig. 3C) compared with PBS-challenged mice, suggesting the presence of a procoagulant state in this model. The levels of TAT in *S. pneumoniae*–infected mice decreased over time, suggesting that maximal coagulation activation occurred early following infection.

### PAR-1 promotes neutrophil recruitment and alveolar leak following *S. pneumoniae* infection

**FIGURE 4.**
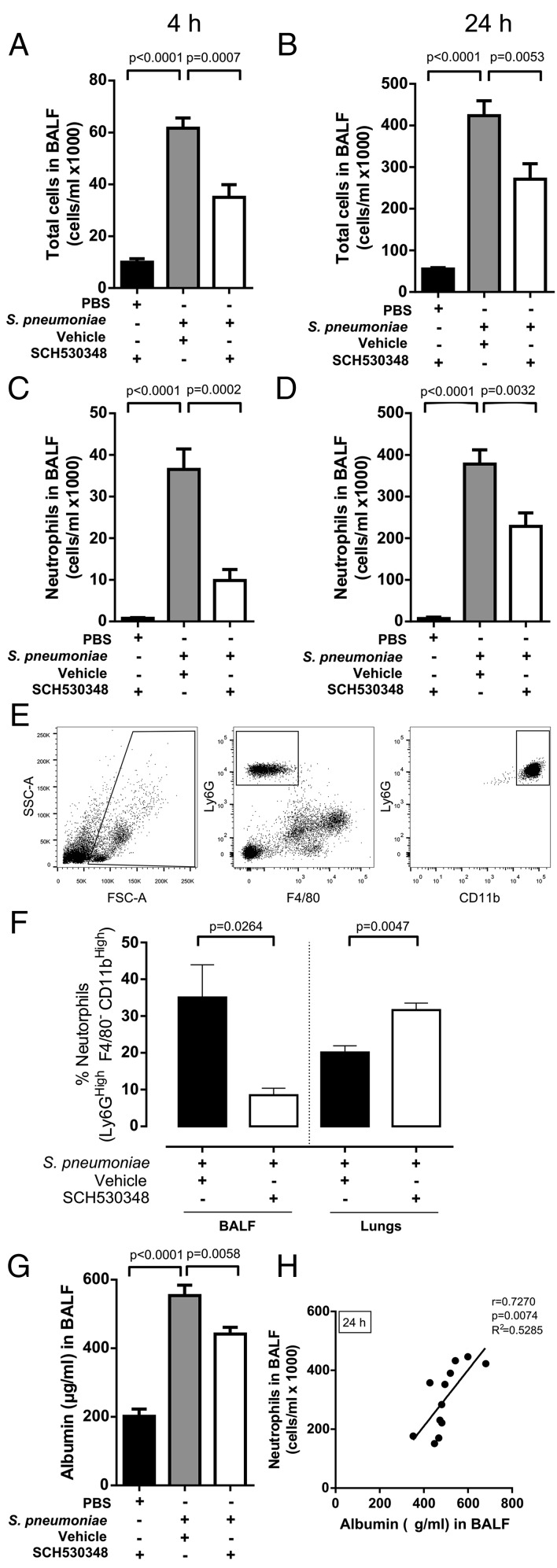
Neutrophil recruitment to the bronchoalveolar space and alveolar barrier disruption are attenuated by PAR-1 antagonism following *S. pneumoniae* infection. BALB/c mice (7–8 wk old) were challenged intranasally with PBS or *S. pneumoniae* D39 (5 × 10^6^ CFU) and treated with either vehicle or the PAR-1 antagonist. Total cells recovered from BAL fluid at (**A**) 4 and (**B**) 24 h following infection and the total number of neutrophils recovered from BAL fluid were calculated (**C**) 4 and (**D**) 24 h following infection. (**E**) Representative flow cytometry gating for the identification of neutrophils (Ly6G^hi^F4/80^−^CD11b^hi^) and (**F**) proportion of neutrophils identified in BAL fluid and whole-lung homogenates at 4 h following infection in mice treated with the PAR-1 antagonist or vehicle. Furthermore, (**G**) BAL fluid albumin levels were measured by ELISA from recovered BAL fluid at 24 h postchallenge. Data are expressed as bar graphs with means ± SEM (*n* = 4–6/group) and were analyzed with one-way ANOVA with a Holm–Sidak post hoc test. Scatter plot is shown with linear regression line for neutrophil counts and albumin levels in (**H**) BAL fluid at 24 h for mice challenged with *S. pneumoniae* and treated with vehicle or the PAR-1 antagonist; data were analyzed with a Pearson correlation coefficient and linear regression.

The potential functional role of PAR-1 in *S. pneumoniae*–induced neutrophil recruitment was next investigated by treating mice with the PAR-1 antagonist SCH530348 (10 mg/kg, i.p.) or drug vehicle alone. Total BAL fluid cell counts increased by 6- and 8-fold in *S. pneumoniae*–infected mice at 4 and 24 h, respectively. PAR-1 antagonist treatment was associated with a significant reduction in BAL fluid total cell counts (by ∼43%) and neutrophil counts (∼73%) at 4 h, compared with drug vehicle-treated mice (Fig. 4A, 4C). The therapeutic effect of SCH530348 was maintained at 24 h (Fig. 4B, 4D), but the magnitude of the inhibitory effect was reduced at this time point. The effect of PAR-1 antagonism on neutrophil recruitment to airspaces was confirmed in the same model using a second small molecule PAR-1 antagonist, RWJ58259 (Supplemental Fig. 1), and in mice infected with a less invasive *S. pneumoniae* strain, EF3030 (Supplemental Fig. 2). PAR-1 antagonism in mice infected with *S. pneumoniae* EF3030 was associated with a reduction in neutrophil counts in the airspaces at 4 and 24 h, but this reduction was only statistically significant at 4 h. Taken together, these data led us to conclude that PAR-1 contributes to the recruitment of neutrophils into the alveolar space during *S. pneumoniae* pneumonia. Additionally, we determined the percentage of neutrophils (Ly6G^hi^F4/80^−^CD11b^hi^ cells) in the BAL fluid and whole-lung homogenates of mice treated with the PAR-1 antagonist or vehicle at 4 h following *S. pneumoniae* challenge (Fig. 4E, 4F). PAR-1 antagonism resulted in a significant reduction in the proportion of neutrophils recruited to the airspaces, whereas the percentage of neutrophils in whole-lung homogenates increased compared with vehicle-treated mice, suggesting that PAR-1 antagonism resulted in neutrophil retention in the pulmonary vasculature or lung parenchyma.

We next investigated the role of PAR-1 in regulating alveolar barrier function during *S. pneumoniae* infection by measuring BAL fluid albumin levels. BAL fluid albumin levels were significantly increased 24 h following *S. pneumoniae* infection. This increase was significantly reduced by PAR-1 antagonism (Fig. 4E). Moreover, there was a strong positive correlation (*r* = 0.7270, *R*^2^ = 0.5285, *p* = 0.0074) between BAL fluid neutrophil counts and BAL fluid albumin levels (Fig. 4F), further supporting the conclusion that neutrophil recruitment is associated with increased alveolar leak during *S. pneumoniae* infection.

### PAR-1 regulates early inflammatory cytokines and chemokines

**FIGURE 5.**
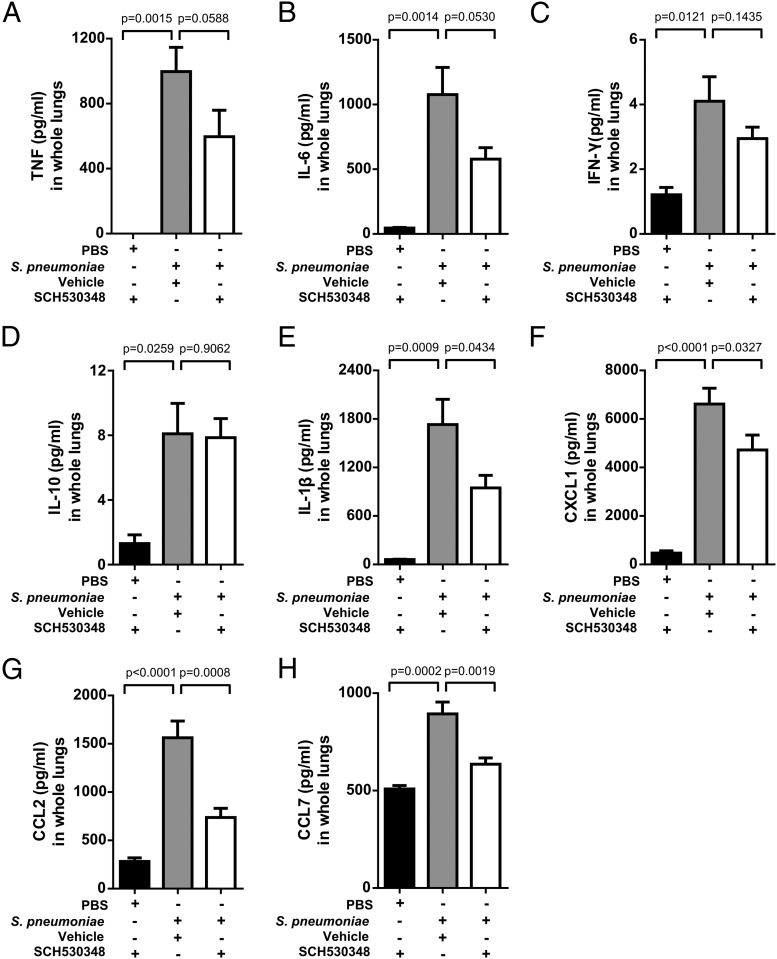
PAR-1 antagonism reduces inflammation in *S. pneumoniae* infection. BALB/c mice (7–8 wk old) were challenged intranasally with PBS and or *S. pneumoniae* D39 (5 × 10^6^ CFU), treated with either vehicle or the PAR-1 antagonist, and culled at 4 h. (**A**–**F**) Whole-lung homogenate levels of TNF, IL-6, IL-1β, IL-10, IFN-γ, and CXCL1 measured by multiplex ELISA (Meso Scale Discovery) and (**G** and **H**) whole-lung homogenate levels of CCL2 and CCL7 measured by ELISA. Data are expressed as means ± SEM (*n* = 4–6/group). Data were analyzed with one-way ANOVA with a Holm–Sidak post hoc test.

To delineate the potential mechanism by which PAR-1 influences neutrophil recruitment during *S. pneumoniae* infection, we measured the pulmonary levels of several key proinflammatory cytokines and chemokines at 4 h. This time point was chosen on the basis that this is the time point at which TAT levels are maximally increased in this model and at which the PAR-1 antagonist exerted its maximal inhibitory effect on neutrophil recruitment. The levels of TNF and IL-6 were increased in response to *S. pneumoniae* infection, and although these levels were attenuated in response to PAR-1 antagonist treatment, this was not statistically significant (Fig. 5A, 5B). The increase in the levels of IFN-γ and IL-10 were not affected (Fig. 5C, 5D). In contrast, PAR-1 antagonist treatment significantly attenuated the elevated lung levels of the canonical proinflammatory cytokine IL-1β (Fig. 5E) and the chemokines CXCL1, CCL2, and CCL7 (Fig. 5F–H) following *S. pneumoniae* infection. Taken together, these data suggest that PAR-1 may influence neutrophil recruitment by regulating the levels of several proinflammatory cytokines and chemokines.

### IL-1β, CXCL1, and CCL7 mediate neutrophil recruitment to the airspaces in different pulmonary compartments

**FIGURE 6.**
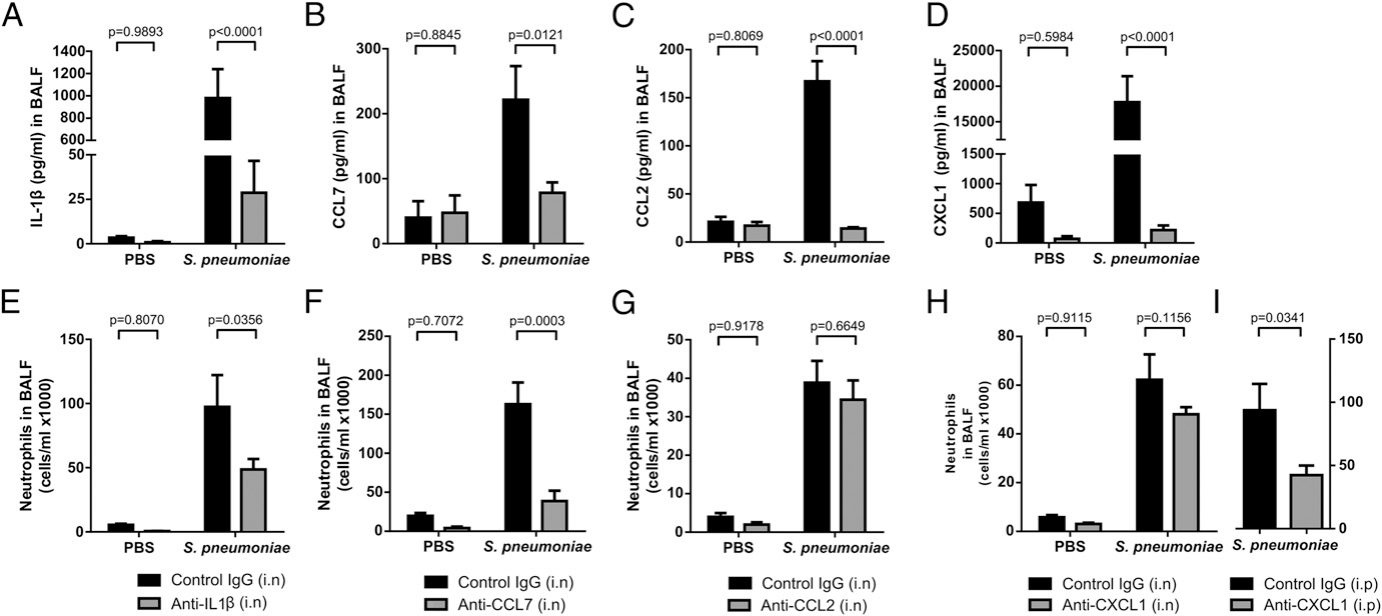
Neutralization of broncholaveolar CCL7 and IL-1β in the alveolar compartment and systemic neutralization of anti-CXCL1 reduce neutrophil recruitment to the bronchoalveolar space. BALB/c mice (7–8 wk old) were challenged intranasally with 10 μg neutralizing Ab (anti–IL-1β, anti-CCL7, anti-CCL2, and anti-CXCL1) and PBS or *S. pneumoniae* D39 (5 × 10^6^ CFU) and culled at 4 h. (**A**–**D**) BAL fluid was recovered and the levels of IL-1β, CCL7, CCL2, and CXCL1 were measured. Neutrophil counts in BAL fluid were determined from cytospin preparations and total cells counts for mice treated intranasally with (**E**) anti–IL-1β, (**F**) anti-CCL7, (**G**) anti-CCL2, and (**H**) anti-CXCL1 and from mice treated with (**I**) anti-CXCL1 by i.p. injection. Data are expressed as bar graphs with means ± SEM (*n* = 5–6/group) and were analyzed with two-way ANOVA with a Holm–Sidak post hoc test.

We next assessed the contribution of IL-1β, CXCL1, CCL2, and CCL7 to the recruitment of neutrophils during *S. pneumoniae* pneumonia using specific neutralizing Abs administered intranasally. Assessment of BAL fluid cytokine levels confirmed that this Ab neutralization strategy resulted in nearly complete neutralization of each of these mediators in response to *S. pneumoniae* infection (Fig. 6A–D). Intra-alveolar neutralization of either IL-1β or CCL7 resulted in significant reductions (∼52 and ∼77%, respectively) in the number of neutrophils recruited to the airspaces in *S. pneumoniae*–challenged mice (Fig. 6E, 6F). In contrast, intra-alveolar neutralization of either CXCL1 or CCL2 had no effect (Fig. 6G, 6H).

CXCL1 is a major classical neutrophil chemoattractant in mice (23). We therefore also examined the effect of the anti-CXCL1 Ab given via systemic delivery (i.p. injection). Systemic neutralization of CXCL1 resulted in a 55% reduction (*p* = 0.0341) in neutrophil accumulation in the airspaces in response to *S. pneumoniae* infection (Fig. 6I), demonstrating a potential role for this chemokine within the vascular compartment.

Finally, to rule out the potential possibility that the attenuated neutrophil responses in mice treated with either anti–IL-1β, anti-CXCL1, or and anti-CCL7 Abs might be related to a reduction in bacterial load, we also measured the BAL and lung *S. pneumoniae* CFU. These were found to be similar for anti-CXCL1–, anti-CCL7–, and anti-CCL2 –treated mice (Supplemental Fig. 3A–C, 3E), or even slightly higher in the case of anti–IL-1β-treated mice (Supplemental Fig. 3D) compared with mice given IgG control Abs. Hence, a reduction in bacterial load is unlikely to explain the reduction in neutrophil accumulation observed in Ab-treated mice.

### PAR-1 antagonism does not compromise host defense

**FIGURE 7.**
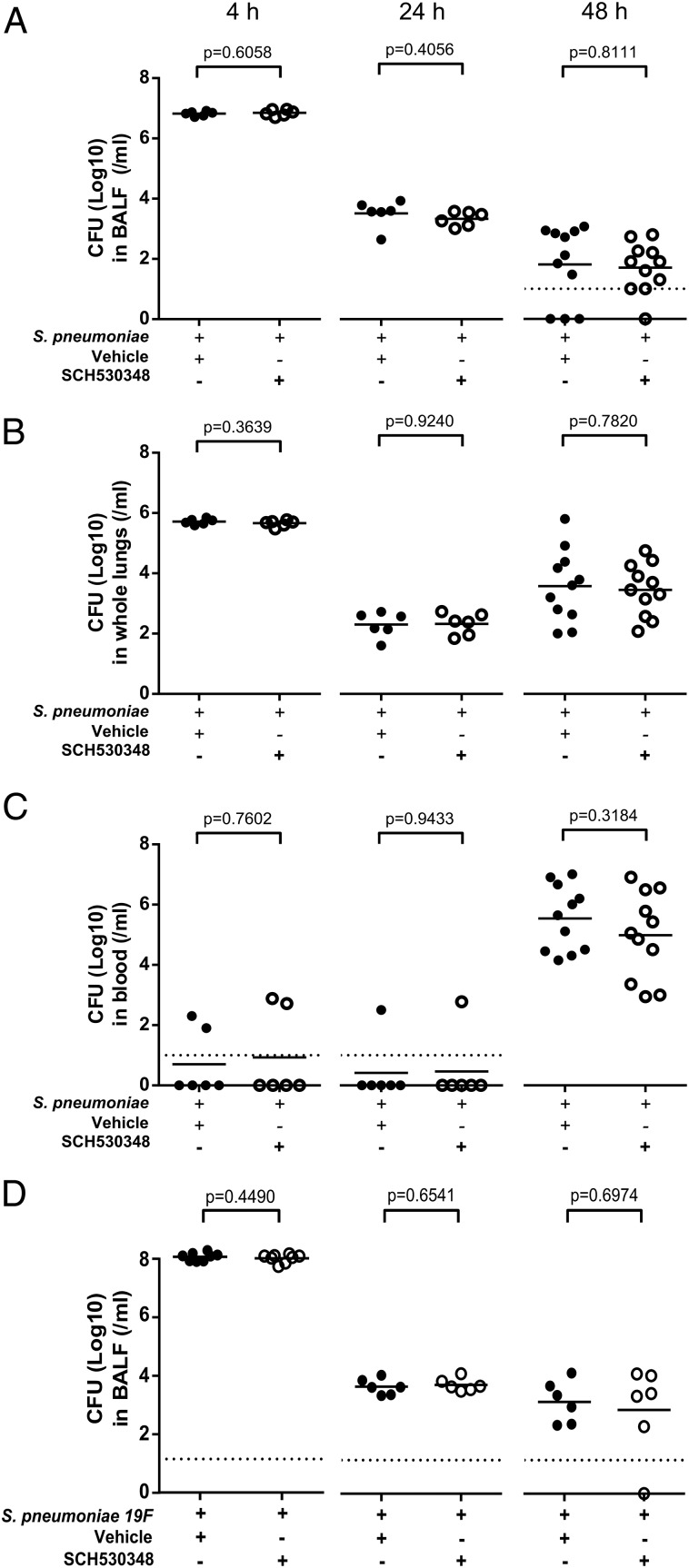
PAR-1 antagonism with SCH530348 does not compromise host defense. BALB/c mice (7–8 wk old) were challenged intranasally with PBS and treated with vehicle or challenged with *S. pneumoniae* D39 or *S. pneumoniae* EF3030 (5 × 10^6^ CFU) and treated with either vehicle or the PAR-1 antagonist. Mice were culled at 4, 24, or 48 h. Bacteria were cultured from recovered (**A**) BAL fluid, (**B**) whole-lung homogenates, and (**C**) blood at 4, 24, and 48 h, respectively, for mice challenged with D39 and (**D**) BAL fluid at 4, 24, and 48 h for mice challenged with EF3030. Data are expressed as dot plots with mean (*n* = 6–8/group) and analyzed with a Student *t* test.

Having demonstrated that PAR-1 influences neutrophil recruitment by regulating the pulmonary levels of several cytokines and chemokines, we next evaluated the effect of PAR-1 antagonism on host defense by measuring *S. pneumoniae* CFU recovered from BAL fluid (Fig. 7A), lung homogenates (Fig. 7B), and blood (Fig. 7C). The CFU recovered in BAL fluid and lung homogenates was highest at 4 h and declined at 24 and 48 h. In contrast, blood CFU increased over time. At all time points examined, we found that there were no differences in BAL fluid, lung, and blood *S. pneumoniae* CFU between mice treated with the PAR-1 antagonist or the drug vehicle (4, 24, and 48 h; Fig. 7A–C), indicating that antagonizing PAR-1 reduces neutrophil recruitment but does not appear to compromise the ability of the host to control bacterial outgrowth. This finding was further confirmed using a second PAR-1 antagonist (RWJ58259) (Supplemental Fig. 1). The effect of PAR-1 antagonism on host defense was also assessed using the EF3030 *S. pneumoniae* noninvasive pneumonia model (Fig. 7D), which has previously been shown to cause bacteremia when host defense is compromised (10, 24). Treatment with SCH530348 did not result in increased recovery of EF3030 *S. pneumoniae* CFU from BAL fluid at 4, 24, and 48 h. Additionally, mice did not develop bacteremia at any of these time points, indicating that despite the reduction in neutrophil recruitment, PAR-1 antagonism did not lead to invasive infection with this pathogen.

## Discussion

Infection with *S. pneumoniae* is characterized by a pronounced neutrophilic inflammatory response, an intra-alveolar procoagulant state, and disruption of the alveolar epithelial barrier leading to the leakage of protein-rich fluid into the alveolar space and pulmonary edema. However, the extent to which neutrophils contribute to alveolar leak, as well as the interplay between inflammation and coagulation, remains poorly characterized. We now demonstrate that neutrophils are important for controlling bacterial outgrowth, but they also promote alveolar barrier disruption. We further show that neutrophil recruitment is regulated by the major high-affinity thrombin receptor, PAR-1, which is upregulated following infection with *S. pneumoniae*. PAR-1 was shown to regulate neutrophil accumulation by modulating the expression of several proinflammatory cytokines, including IL-1β and the chemokines CXCL1 and CCL7. Although the control of bacterial outgrowth is dependent on neutrophils, modulating excessive neutrophilic inflammation by antagonizing PAR-1, or neutralizing IL-1β, CXCL1, or CCL7, attenuates neutrophil recruitment without affecting bacterial outgrowth. Taken together, these studies reveal a previously unidentified mechanism that mediates neutrophilic inflammation during pulmonary bacterial infection.

Neutrophil recruitment into the lung is a multistep process involving extravasation across the pulmonary endothelium, trafficking across the lung interstitium, and migration across the alveolar epithelium into airspaces. This involves multiple chemotactic molecules acting at specific spatiotemporal checkpoints (23). The microanatomy of the distal lung provides an unusual environment for neutrophil migration, as sheer forces are low in the narrow pulmonary capillary network so that neutrophils do not undergo the classical rolling, slowing, and adhesion associated with extravasation observed in other organs (23, 25). Furthermore, it has been estimated that the pool of neutrophils sequestered within the pulmonary microvasculature exceeds the size of the circulating pool by ∼5-fold so that there is a large pool of intrapulmonary neutrophils that can be rapidly mobilized in response to inflammatory insults and infection (23). However, the contribution of neutrophils to barrier disruption during lung inflammation remains enigmatic, and current evidence suggests that it is highly dependent on both the nature and location of the primary insult (23). In vitro studies employing lung epithelial cells support the notion that neutrophils are capable of promoting epithelial barrier disruption (26–28). This has also been confirmed in animal models of pancreatitis-associated lung injury (29), lung reperfusion injury (30), LPS-induced lung injury (31), and in the context of hantavirus infection (32). However, other animal models of lung injury suggest that alveolar barrier disruption is independent of neutrophil recruitment (33–35). Both the contribution of neutrophils to alveolar leak and the mechanisms responsible for neutrophil recruitment in the context of bacterial infection still remain poorly defined. We now report that nearly complete neutrophil depletion significantly attenuated alveolar leak during *S. pneumoniae* lung infection. To the best of our knowledge, this represents the first in vivo evidence linking neutrophil recruitment to disruption of the alveolar barrier during pulmonary *S. pneumoniae* infection. However, keep in mind that nearly complete neutrophil depletion compromised the ability of the host to control bacterial outgrowth (10). These observations are in line with the notion that the recruitment of neutrophils to sites of lung infection needs to be finely balanced so as to provide adequate host defense without promoting bystander tissue damage.

The local intravascular activation of the coagulation cascade is part of the normal response to lung injury and ensures that there is limited blood loss in response to damage to the extensive lung microvasculature. Additionally, there is evidence that tissue factor–dependent coagulation within the intra-alveolar compartment also promotes lung inflammatory and fibroproliferative responses in the settings of acute and chronic lung injury via both fibrinogen-dependent and fibrinogen-independent cellular responses (36–38). Current evidence supports a role for the major high-affinity thrombin-receptor, PAR-1, in mediating the interplay between coagulation, inflammation, and tissue repair in both the lung and other organs (15, 39). In uninjured lungs, PAR-1 is expressed by multiple cell types, including alveolar macrophages, dendritic cells, fibroblasts, bronchial and alveolar epithelium, and pulmonary endothelium (14). Additionally, it is expressed on monocytes and lymphocytes recruited to the lungs, but it is not expressed by neutrophils (14). In the present study, we now report that PAR-1 immunostaining is increased during *S. pneumoniae* pneumonia, most obviously in the endothelium and epithelium. The regulation of PAR-1 expression in the lung is not fully understood; however, a role for Rac1 in regulating surface expression of this receptor in vascular smooth muscle cells has been identified (40), whereas the transcription factors AP-2, specificity protein-1, early growth response-1, and the tumor suppressor gene p53 have been shown to regulate PAR-1 gene expression on numerous cell types (41, 42).

We now report that PAR-1 antagonism with the recently U.S. Food and Drug Administration–approved (43), highly potent, and selective PAR-1 antagonist SCH530348 greatly attenuated pulmonary neutrophil recruitment to the airspaces of *S. pneumoniae*–infected mice. This finding was confirmed using a second structurally unrelated selective PAR-1 antagonist, RWJ58259. Furthermore, the reduction in neutrophil recruitment to airspaces was associated with neutrophil retention in the pulmonary vasculature or lung parenchyma, suggesting that antagonism of PAR-1 prevents neutrophil transmigration across the endothelium or alveolar epithelium. The exact mechanism remains to be elucidated, but PAR-1 has been shown to regulate the expression of endothelial cell adhesion molecules, such as ICAM-1 and VCAM-1, which in turn may influence the migration of neutrophils into different tissue microcompartments within the lung (44). PAR-1 antagonism was also associated with a significant reduction in alveolar leak, again suggesting that neutrophils contribute to barrier disruption. Also, bear in mind that the attenuation in alveolar leak in PAR-1 antagonist-treated mice might be a direct effect of PAR-1 on endothelial cells (45). PAR-1 plays an important role in regulating endothelial barrier integrity and, depending on the nature and concentration of the activating proteinase, it can be either barrier disruptive or barrier-protective (14). Indeed, low thrombin concentrations and activated protein C acting on PAR-1 can lead to transactivation of the barrier-protective sphingosine-1 phosphate receptor-1 (46, 47), whereas high thrombin concentrations and matrix metalloproteinase-1, also acting via PAR-1, increase vascular permeability through activation of sphingosine-1 phosphate receptor-3 (48). Furthermore, the beneficial role of PAR-1 in a model of lethal sepsis (induced in response to bacterial peritonitis by cecal ligation and puncture) has been shown to switch during the progression of sepsis. Whereas early blockade of PAR-1 improves survival, late blockade was found to be detrimental to the host (49). In our model of *S. pneumoniae*–induced pneumonia, the beneficial effect of blocking PAR-1 leads to a prolonged reduction in neutrophil recruitment and alveolar leak, suggesting that the role of PAR-1 in mediating inflammation and barrier disruption is indeed highly context-dependent.

Additionally, note that the mode of PAR-1 inhibition might also dictate outcome, as has been previously reported in the context of cardiac remodeling and hypertrophy (50). In the present study, we elected to investigate the role of PAR-1 following *S. pneumoniae* infection by using a potent and highly selective PAR-1 antagonist SCH530348 (18, 19), which has recently been approved by the U.S. Food and Drug Administration for the treatment and prevention of atherothrombotic events in postmyocardial infarction patients (43). Our study revealed that PAR-1 inhibition resulted in an early (4 h) and late (24 h) inhibition of neutrophil recruitment and lower levels of proinflammatory mediators following challenge with *S. pneumoniae* with no deleterious effects on bacterial outgrowth. This is in contrast to observations reported for *S. pneumoniae* challenge in PAR-1 knockout (PAR-1^−/−^) mice, which exhibit lower bacterial loads, enhanced proinflammatory mediator levels, and only a late attenuation of neutrophil recruitment (51). In terms of likely explanations for these differences, it is possible that potential compensatory pathways, including signaling via other PARs in PAR-1^−/−^ mice, as has been previously suggested in the context of embryonic development (52) and cardiac remodeling in PAR-1–deficient animals (50), might be critical. Differences in mouse (BALB/c versus C57BL/6) and bacterial background strain (serotype 2 versus 3) could also provide a potential explanation.

Because PAR-1 antagonism abrogated the recruitment of neutrophils into lung airspaces during *S. pneumoniae* infection, we next sought to determine the potential role of PAR-1 in regulating proinflammatory mediator levels. TNF and IL-1 are considered to be two key early proinflammatory response cytokines induced following infection. For example, previous studies using TNF knockout mice have shown reduced neutrophil recruitment following *S. pneumoniae* infection (53). We now demonstrate that PAR-1 antagonism only had a modest effect on TNF levels but that it significantly reduced the expression of IL-1β. Furthermore, neutralization of IL-1β resulted in reduced neutrophil accumulation, suggesting that IL-1β is a key cytokine involved in modulating the recruitment of neutrophils downstream of PAR-1. Indeed, previous studies have demonstrated a role for IL-1β signaling in the recruitment of neutrophils in response to *S. pneumoniae* (53, 54).

In vitro studies in diverse cell types have demonstrated that PAR-1 is capable of engaging multiple signaling cascades and thereby influences a variety of cellular responses, including regulating the expression and release of inflammatory cytokines and chemokines (14, 55, 56). In the present study, we provide evidence that PAR-1 antagonism also significantly reduced levels of CXCL1, CCL2, and CCL7 in response to *S. pneumoniae* infection. Moreover, intra-alveolar neutralization of the non–classical neutrophil chemoattractant CCL7 attenuated neutrophil recruitment to alveolar airspaces. However, intra-alveolar neutralization of the related chemokine CCL2, as well as the classical neutrophil chemoattractant CXCL1, had no effect, despite evidence of effective neutralization of these chemokines. The lack of an effect of anti-CXCL1 on neutrophil recruitment was unexpected given that previous studies using anti-CXCL1 Abs or CXCR2 knockout mice support a role for this chemokine in neutrophil recruitment during *S. pneumoniae* infection (10, 57). Indeed, in our model, subsequent studies revealed that the route of Ab administration was a critical determinant of outcome in that systemic neutralization by i.p. injection of anti-CXCL1 was found to be highly effective at reducing neutrophil recruitment to airspaces. Taken together, these data suggest that CXCL1 in the pulmonary vasculature, rather than the local intra-alveolar compartment, might be important for pulmonary neutrophil transmigration in this model. Furthermore, CXCL1 and CCL7 may differentially influence neutrophil migration within discrete pulmonary compartments, such as the endothelium, interstitium, and epithelium. However, these studies highlight the contextual complexities of the pulmonary chemokine network and the spatial and temporal compartmentalization within the lung (23). Finally, the dynamic nature of host–pathogen interactions also means that inherent interexperimental variation can be a factor in animal models of bacterial infection, and therefore any resulting data should be interpreted with this in mind.

Our study also addressed the potential contribution of neutrophilic inflammation to control bacterial outgrowth. Bacterial CFU recovered in BAL fluid and whole-lung homogenates was highest at 4 h and declined at 24 and 48 h. The 4 h bacterial CFU likely represents the initial bacterial inoculum. Bacterial CFU then decreases by 24 h as the inoculated bacteria are cleared by the host immune response. Nearly complete depletion of the neutrophil population severely compromised host defense, indicating that bacterial clearance is dependent on neutrophil recruitment into the airspaces. Therefore, it was important for us to evaluate the effect of PAR-1 antagonist treatment on bacterial clearance, considering the significant reduction, but not complete abrogation, of neutrophil numbers recovered in BAL fluid. Our data revealed that the attenuated neutrophil response following PAR-1 antagonist treatment was sufficient for the control of bacterial outgrowth with both invasive and noninvasive strains of *S. pneumoniae*. This suggests that reducing excessive neutrophilic inflammation does not necessarily impair the ability of the host to control infection and that only a small proportion of recruited neutrophils might be needed to provide adequate protection.

Additionally, in experimental lung injury, airspace neutrophil accumulation has been associated with several physiological parameters associated with disease severity (e.g., arterial oxygen saturation, respiratory rate, and heart rate) (12), suggesting that attenuating neutrophil recruitment to the lungs without compromising host defense could be clinically beneficial. This may be particularly relevant in adult respiratory distress syndrome and other pulmonary conditions associated with bacterial infection, including exacerbation of chronic obstructive pulmonary disease, where bacterial infection may be controlled by the use of antibiotics. Strategies aimed at controlling neutrophilic inflammation and the resultant lung injury may therefore represent potential complementary treatment options.

In summary, the data reported in this study suggest a key role for PAR-1 in the innate immune response to *S. pneumoniae* infection. PAR-1 expression is increased in the lungs following challenge to *S. pneumoniae* and contributes to the neutrophilic response by influencing multiple downstream inflammatory mediators, including IL-1β, CXCL1, and CCL7 within different lung compartments. Moreover, PAR-1 antagonism attenuated alveolar barrier disruption without impairing host defense. Finally, although further studies are needed to fully understand the role of this receptor in different pneumonia models, to our knowledge, the present study provides the first preclinical proof of concept that PAR-1 antagonism may represent a potential novel therapeutic approach for the prevention of excessive neutrophilic lung injury associated with bacterial pneumonia.

## Supplementary Material

Data Supplement
