# Supplementary material for: Regulation of Neutrophilic Inflammation by Proteinase-Activated Receptor 1 during Bacterial Pulmonary Infection
Source: J Immunol. 2015 May 6;194(12):6024–34. doi: 10.4049/jimmunol.1500124 (PMC4456635; doi:10.4049/jimmunol.1500124)
Supplement: Data Supplement [file JI_1500124.zip › JI_1500124_Supplemental_Figures_1.pdf]

ONLINE SUPPLEMENTARY FILE

Regulation of neutrophilic inflammation by proteinase-activated receptor-1 during bacterial pulmonary infection

R. J. José, A. E. Williams, P. F. Mercer, M. G. Sulikowski, J. S. Brown, R. C. Chambers

Centre for Inflammation and Tissue Repair, University College London, London, United Kingdom

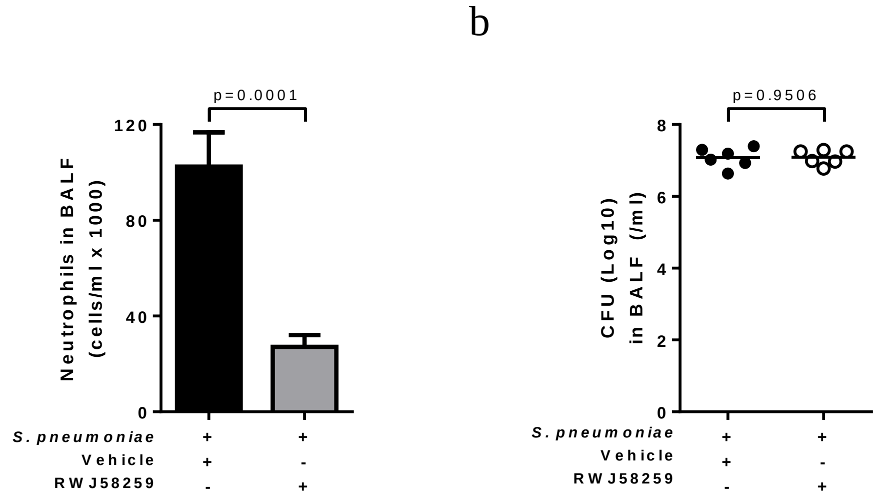

Figure 1: **Effect of RWJ58259 on BAL fluid cell counts and CFU following *S. pneumoniae* infection**

BALB/c mice (7-8 weeks old) were challenged intra-nasally with *S. pneumoniae* (D39, $5x10^6$ CFU and immediately treated with RWJ58259 (5 mg/kg) or vehicle (11% DMSO), and culled 3 h post-challenge. Data are expressed as bar graphs with mean ± SEM (n=12 per group) for (a) BAL fluid neutrophil and (b) BAL fluid CFU data are expressed as dot plots (n=6 per group). Data were analysed with Student's t-test.

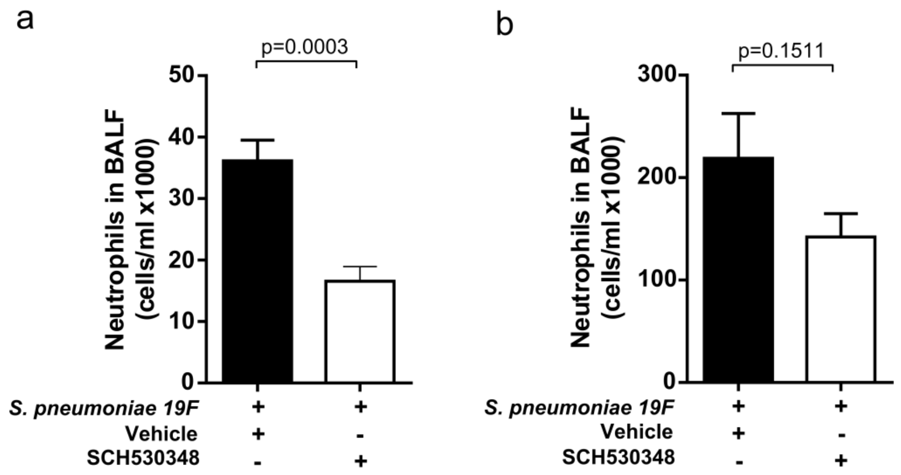

Figure 2: SCH530348 attenuates neutrophil recruitment to airspaces following challenge *with S. pneumoniae* EF3030. BALB/c mice (7-8 weeks old) were challenged intra-nasally with *S. pneumoniae* 19F (5×10^6 CFU) and treated with either vehicle or the PAR-1 antagonist. The total number of neutrophils recovered from BAL fluid were calculated (**a**) 4 h and (**b**) 24 h following infection Data are expressed as bar graphs with mean ± SEM (n=6-8 per group) and analysed with Student's t-test.

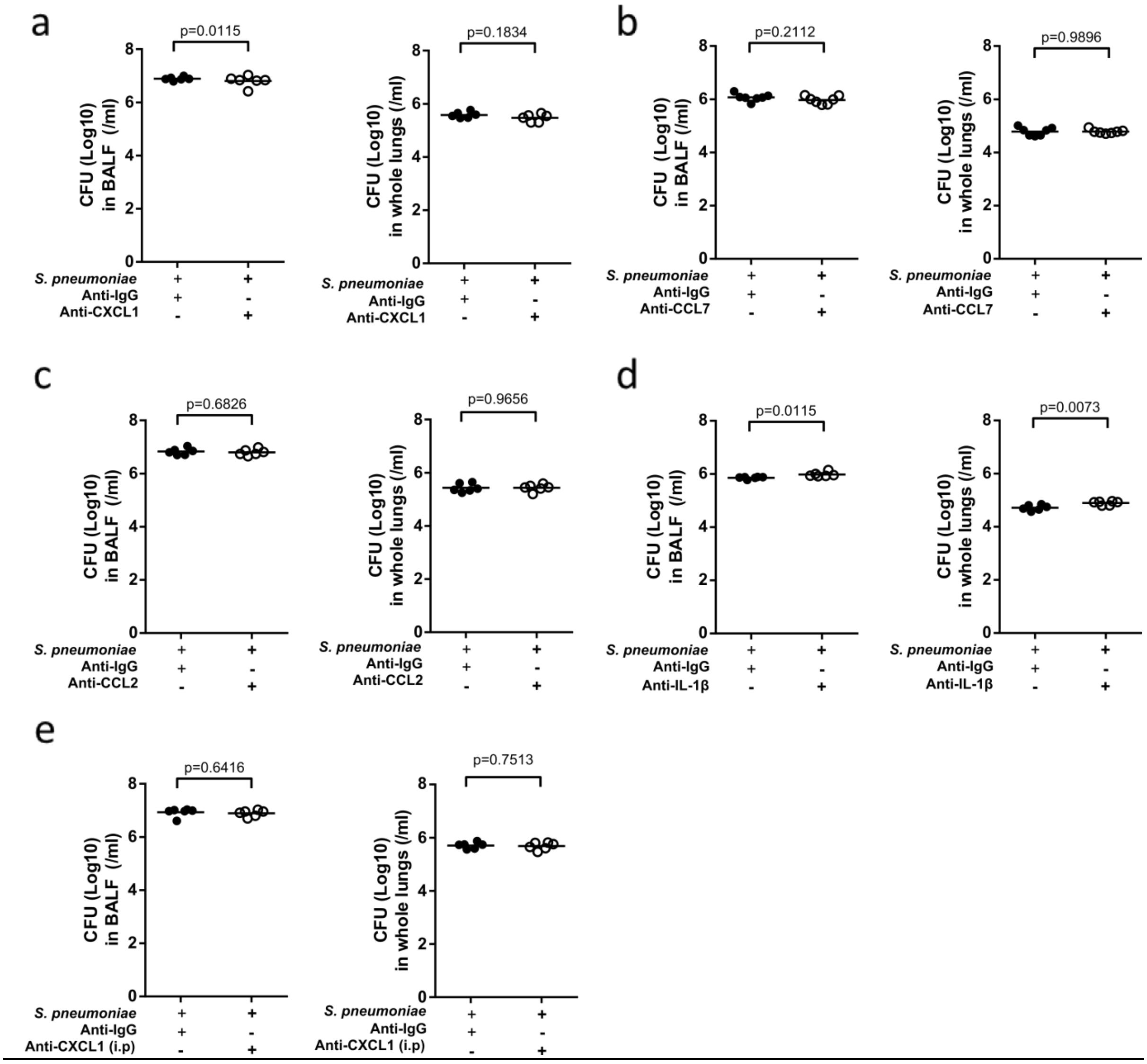

Figure 3: Effect of intra-nasal anti-IL1β, anti-CCL7, anti-CCL2 and anti-CXCL1, and the effect of intra-peritoneal anti-CXCL1 on bacterial CFU

BALB/c mice (7-8 weeks old) were challenged intra-nasally with 10 µg of neutralising antibody (either anti-CXCL1 (A), anti-CCL7 (B), anti-CCL2 (C) or anti-IL-1β(D)) or intra-peritoneally with anti-CXCL1 (E) and *S. pneumoniae* (D39, 5x10^{6} CFU), and culled at 4 h. Data are expressed as dot plots with mean (n=6 per group) for CFU recovered from BALF and whole lungs. Data were analysed with Student's t-test
